# Influence of homoarginine on creatine accumulation and biosynthesis in the mouse

**DOI:** 10.3389/fnut.2022.969702

**Published:** 2022-08-09

**Authors:** Craig A. Lygate, Hannah A. Lake, Debra J. McAndrew, Stefan Neubauer, Sevasti Zervou

**Affiliations:** Division of Cardiovascular Medicine, Radcliffe Department of Medicine and British Heart Foundation Centre for Research Excellence, University of Oxford, Oxford, United Kingdom

**Keywords:** creatine, homoarginine, L-arginine:glycine amidinotransferase (AGAT), guanidinoacetate N-methyltransferase (GAMT), creatine transporter, pancreas

## Abstract

Organisms obtain creatine from their diet or by *de novo* synthesis *via* AGAT (L-arginine:glycine amidinotransferase) and GAMT (Guanidinoacetate N-methyltrasferase) in kidney and liver, respectively. AGAT also synthesizes homoarginine (hArg), low levels of which predict poor outcomes in human cardiovascular disease, while supplementation maintains contractility in murine heart failure. However, the expression pattern of AGAT has not been systematically studied in mouse tissues and nothing is known about potential feedback interactions between creatine and hArg. Herein, we show that C57BL/6J mice express AGAT and GAMT in kidney and liver respectively, whereas pancreas was the only organ to express appreciable levels of both enzymes, but no detectable transmembrane creatine transporter (*Slc6A8*). In contrast, kidney, left ventricle (LV), skeletal muscle and brown adipose tissue must rely on creatine transporter for uptake, since biosynthetic enzymes are not expressed. The effects of creatine and hArg supplementation were then tested in wild-type and AGAT knockout mice. Homoarginine did not alter creatine accumulation in plasma, LV or kidney, whereas in pancreas from AGAT KO, the addition of hArg resulted in higher levels of tissue creatine than creatine-supplementation alone (*P* < 0.05). AGAT protein expression in kidney was downregulated by creatine supplementation (*P* < 0.05), consistent with previous reports of end-product repression. For the first time, we show that hArg supplementation causes a similar down-regulation of AGAT protein (*P* < 0.05). These effects on AGAT were absent in the pancreas, suggesting organ specific mechanisms of regulation. These findings highlight the potential for interactions between creatine and hArg that may have implications for the use of dietary supplements and other therapeutic interventions.

## Introduction

Creatine plays an important role in the storage and buffering of energy in excitable cells such as cardiac and skeletal myocytes, where it is interconverted to phosphocreatine *via* the creatine kinase reaction (1, 2). Creatine can be obtained either from the diet or *via de novo* biosynthesis, but since most cells do not express the biosynthetic enzymes, cellular levels are regulated by a specific plasma membrane creatine transporter (CrT; SLC6A8) (3). Eventually creatine is lost due to spontaneous conversion to creatinine and excreted in the urine at a rate of about 1.7% of the total creatine pool per day (4).

Biosynthesis of creatine occurs in two spatially distinct steps. L-Arginine:glycine amidinotransferase (AGAT) in the kidney transfers an amidino group from L-arginine to glycine to produce the precursor guanidinoacetate (GAA) in addition to ornithine. GAA is then methylated to creatine in the liver by the enzyme Guanidinoacetate N-methyltransferase (GAMT) using S-adenosylmethionine (SAM) as the methyl donor (4–6). More recently it has been recognized that a small but significant level of creatine biosynthesis has been described in the rat pancreas, estimated at ~ 8% of total requirements (7).

AGAT is not only responsible for synthesis of GAA but also of the non-proteinogenic amino acid L-homoarginine (hArg) by catalyzing transfer of the guanidine group from arginine to lysine instead of glycine (8, 9). Homoarginine has no known metabolic role, but has recently garnered interest because low levels of plasma hArg are an independent predictor of mortality in human populations of cerebro- and cardio- vascular disease, including in ischemic stroke (9) sudden cardiac death, heart failure, and acute myocardial infarction (10–13). Furthermore, it appears that hArg is actively involved in modifying pathophysiology rather than simply an epiphenomenon and may therefore have therapeutic potential. For example, homoarginine supplementation reduced cerebral damage in a mouse model of ischaemic stroke (9) and preserved contractile reserve in mice with chronic heart failure (14). In a mouse model of atherosclerosis, hArg treatment did not influence plaque development, but did prolong survival *via* a positive effect on cardiac remodeling and function (15). In addition, hArg mildly reduced blood glucose levels in mice fed a high-fat diet, suggesting potential to improve metabolic health (16).

When mice with whole body deletion of AGAT (AGAT KO) are fed a creatine-free diet, they have a complete absence of creatine, while some residual hArg remains due to incidental synthesis *via* the homologous urea cycle (17). The AGAT KO mouse can therefore serve as a convenient experimental model that allows easy manipulation of creatine and hArg *via* dietary supplementation. For example, creatine-naïve AGAT KO have very low body weight, due to minimal body fat, reduced body water, and skeletal muscle atrophy, which are all rescued by dietary creatine (17–19). It is also notable that recent evidence indicates the importance of creatine in brown adipose tissue (BAT) in controlling thermogenesis and therefore adiposity *via* a “futile creatine cycle” (20, 21). In contrast, AGAT KO mice exhibit deficits in cardiac contractility and relaxation that are rescued by hArg, but not by creatine supplementation (18).

It is striking that so much recent work on the physiological role of creatine and hArg has been performed in the mouse, while most of the data on creatine biosynthesis *via* AGAT and GAMT expression are derived from rat (22–24) and humans. There is therefore a need to determine whether the biosynthetic expression profiles and creatine accumulation patterns as previously described are also applicable to mice.

Furthermore, it is unknown whether circulating hArg levels influence tissue creatine levels. End-product inhibition of AGAT has been described for creatine (5, 25), but whether an equivalent effect exists for hArg is unknown. This is an important consideration since both creatine and hArg may be used therapeutically, so it is prudent to understand whether levels of one metabolite affect levels of the other. Here we define relative expression levels of AGAT, GAMT & CrT in relevant peripheral tissues in normal healthy mice. We then use AGAT KO mice with dietary manipulation of creatine and hArg to explore potential interactions in terms of the tissue accumulation of creatine.

## Methods

### Mouse husbandry, creatine and hArg feeding

All animal experiments were approved by the Committee for Animal Care and Ethical Review at the University of Oxford and comply with the Animals (Scientific Procedures) Act 1986. Mice were maintained in specific pathogen-free conditions using individually ventilated cages on a 12 h night/day cycle with controlled temperature (21°C) and humidity.

C57BL/6J^OlaHsd^ mice (*n* = 8 4F/4M) were purchased from Envigo (Blackthorn UK) at 12 weeks of age and maintained on Teklad Global Diet 2916 (Envigo, Blackthorn UK), which is naturally creatine-free. Two weeks later mice were killed by cervical dislocation and tissues rapidly removed, snap frozen in liquid nitrogen, and stored at -80°C.

AGAT^−/−^ mice on a pure C57BL/6J^OlsHsd^ genetic background were bred in-house and genotyped as previously described (17). These mice have a global deficiency of AGAT protein and therefore very low levels of hArg and undetectable levels of creatine (18). This provides a “blank canvas” for the dietary manipulation of these metabolites. Male wild-type (WT) and AGAT^−/−^ (KO) mice were fed R/M-H complete maintenance diet, which is naturally creatine-free, or the same diet supplemented with 0.5% (w/w) creatine monohydrate (Ssniff, Soest, Germany). Some mice also received L-Homoarginine hydrochloride (hArg, Sigma–Aldrich, UK) added to the drinking water at a concentration of 14 mg/L for 4 weeks to provide the experimental groups shown in **Figure 2A**. These are well established doses and routes of administration that are known to be palatable and to replace or augment tissue levels (18). At 16 weeks of age, animals were killed by cervical dislocation and the following tissues harvested and stored as above: a blood sample was obtained by cardiac puncture, LV, skeletal muscle (soleus and gastrocnemius), brown adipose tissue (BAT), kidney, liver, and pancreas.

### Tissue extraction and biochemistry

The tissue samples were powdered while frozen on dry ice prior to total RNA and total protein extraction as described before (26).

### HPLC

Powdered tissue (*n* = 10–12 per group) was prepared for quantification of creatine by HPLC, and then normalized to protein content using the Lowry method as previously described (27, 28). Total creatine values are reported, which represents the combined values for free creatine and phosphocreatine. For measurement of creatine, all mice were male and harvested at 16 weeks.

### qRT-PCR

All reactions were performed using SYBR Green chemistry and the Bio-Rad One-step iTaq kit (Bio-Rad) in a CFX 96 machine (Bio-Rad). Total RNA input was 1ng/μl for all samples (*n* = 8) and normalization was performed using the ΔΔ^Ct^ method on the CFX manager software (29), a 4-point standard curve from a pool of RNA from different tissues. Commonly used reference genes were tested for stability across mouse tissues (data not shown). These were ribosomal (*36B4/Rplp0, Rpl71l*), hypoxanthine-guanine phosphoribosyltransferase (*Hgprt*) endoplasmic reticulum receptor (*Rer1*) cyclophilin A in addition to 18s rRNA, which was found to be the most stable, in agreement with previous studies (30). The rest of the reference genes showed markedly lower mRNA levels in pancreas that would introduce quantitation bias (data not shown). Table 1 shows nucleotide sequences used as oligos in qRT-PCR experiments. Some oligos were custom-designed using SnapGene (v.6.0.2); all oligos were intron-exon spanning.

**Table 1:** Oligonucleotide sequences of primers used in qRT-PCR.

**Gene name**	**Nucleotide ID**	**Forward sequence (5'-3')**	**Reverse sequence (5'-3')**	**Reference**
*Agat*	NM_025961.5	GTGGAGGTGAAGGCCAATA	CACATCTCTTCGACCTCAG	custom
*Gamt*	AF015887	CCTCCAGAGGGGGCCGGGT	GACGCTGGAAGACCCCATC	(31) (custom)
*Slc6A8*	NM_001142809.1	ACTGTGTGGAGATCTTCCGC	CAGCAAGCTGGTCACATGTG	(32)
*18s rRNA*	NR_003278.3	TCGGAACTGAGGCCATGATT	TTTCGCTCTGGTCCGTCTTG	(30)

### Protein expression

AGAT and GAMT protein were detected in tissue panel (kidney, liver, pancreas, LV, skeletal muscle, BAT from *n* = 6 C57BL/6 mice, as described before (28). Electrophoresis was performed using the Bio-Rad system of Mini-Protean TGX 4–20% gradient gels and followed by semi-dry transfer of proteins on a PVDF membrane, in a Trans-Blot Turbo apparatus. Primary antibody against AGAT was commercially available by Proteintech (rabbit polyclonal) and used at 1:2,000 in PBS/5% skimmed milk powder at 4°C overnight. Anti-GAMT rabbit polyclonal antibody was supplied by Novusbio (NBP2-14036) and used at 1:2,000 overnight, similarly to anti-AGAT. For part of the immunoblotting work, β-tubulin (abcam, 1:5,000) was used as a loading control. All blots were incubated using anti-rabbit IgG at 1:20,000 for 1 h and then signal was detected using chemiluminescence (ECL, GE). For protein normalization purposes, all samples were run on stain-free gels (Bio-Rad) to allow for gel light activation post-electrophoresis and imaging of total protein per well (Image Lab software, Bio-Rad). Finally, AGAT and GAMT protein band signal was normalized over total protein in order to avoid variability in the expression levels of reference proteins between tissues or over β-tubulin.

### Data analysis

Statistics were not applied to the gene and protein expression data in C57BL/6J mouse tissues, since there is inherent variability between tissues in the expression of housekeeping genes for normalization and in protein extraction efficiency. However, this data can be interpreted as an all or nothing response showing which tissues express the genes / proteins of interest. For the creatine and homoarginine supplementation experiment, groups were analyzed by one-way ANOVA followed by multiple comparisons (Prism Graphpad v.8.0). Significance levels were at ^*^P < 0.05, ^**^P < 0.01. ^***^P < 0.001 and ^****^P < 0.0001.

## Results

### Expression of creatine transporter and biosynthetic enzymes

**Figure 1:**
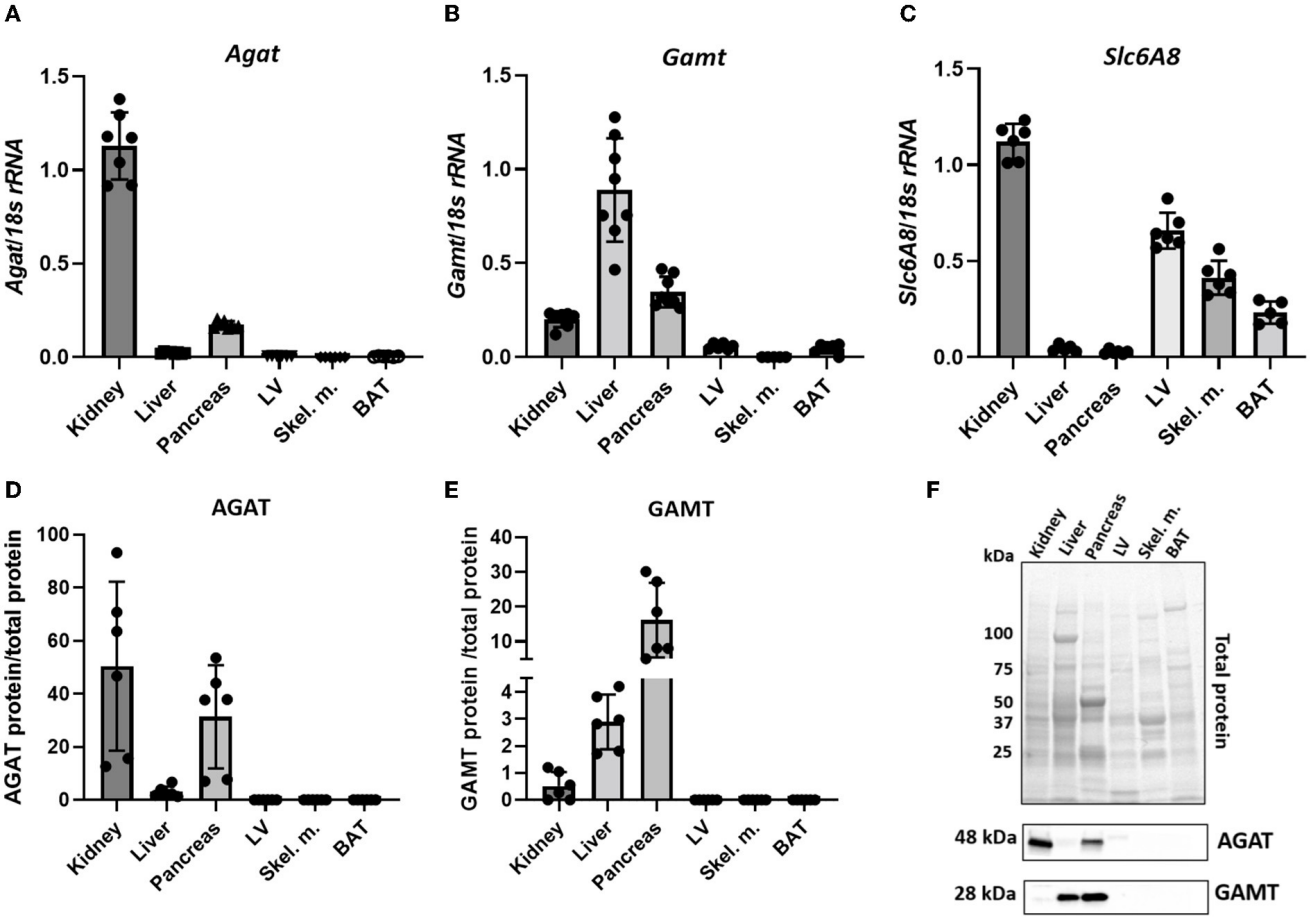
Expression levels of creatine biosynthetic enzymes in a panel of C57BL/6J mouse tissues. **(A,D)** mRNA and protein levels respectively, for AGAT. **(B,E)** mRNA and protein levels, respectively for GAMT. **(C)** mRNA levels for *Slc6A8* (Creatine transporter). (**F)** Representative images from protein electrophoresis showing total protein panel used for normalization purposes, in addition to protein signal detected for AGAT (48 kDa) and GAMT (28 kDa).

Throughout the manuscript L-arginine:glycine amidinotransferase is referred to by its common abbreviation, AGAT (rather than the official gene name GATM), this is in order to avoid confusion with GAMT. *Agat* transcript was detected in kidney and pancreas but not in liver, LV, skeletal muscle or BAT (Figure 1A). The expression pattern was mirrored by protein levels detected by immunoblotting in the same tissues while the immunoblot showed very low levels of AGAT protein in liver (Figures 1D,F). Expression of GAMT mRNA (*Gamt*), the second enzyme in creatine biosynthesis, was pronounced in liver and pancreas with low levels in kidney (Figure 1B). In agreement with transcript expression pattern, GAMT protein, shown by a protein band at 28kDa, is also predominantly expressed in liver and pancreas but absent from LV, skeletal muscle and BAT (Figures 1E,F).

Expression levels of CrT (*Slc6a8*) indicate an ability for cellular creatine uptake. In particular, CrT was found to be predominantly expressed in kidney, LV, skeletal muscle and BAT (Figure 1C). Liver does not express CK (33) and therefore has no need for the creatine it synthesizes, hence liver expression of CrT was negligible. Pancreas was the only tissue to express both biosynthetic enzymes, but did not express CrT, suggesting that it relies predominantly on local *de novo* synthesis of creatine. Protein expression of CrT was not assessed due to the lack of high quality specific antibodies suitable for tissue quantification (34).

### Tissue creatine accumulation in response to dietary manipulation

**Figure 2:**
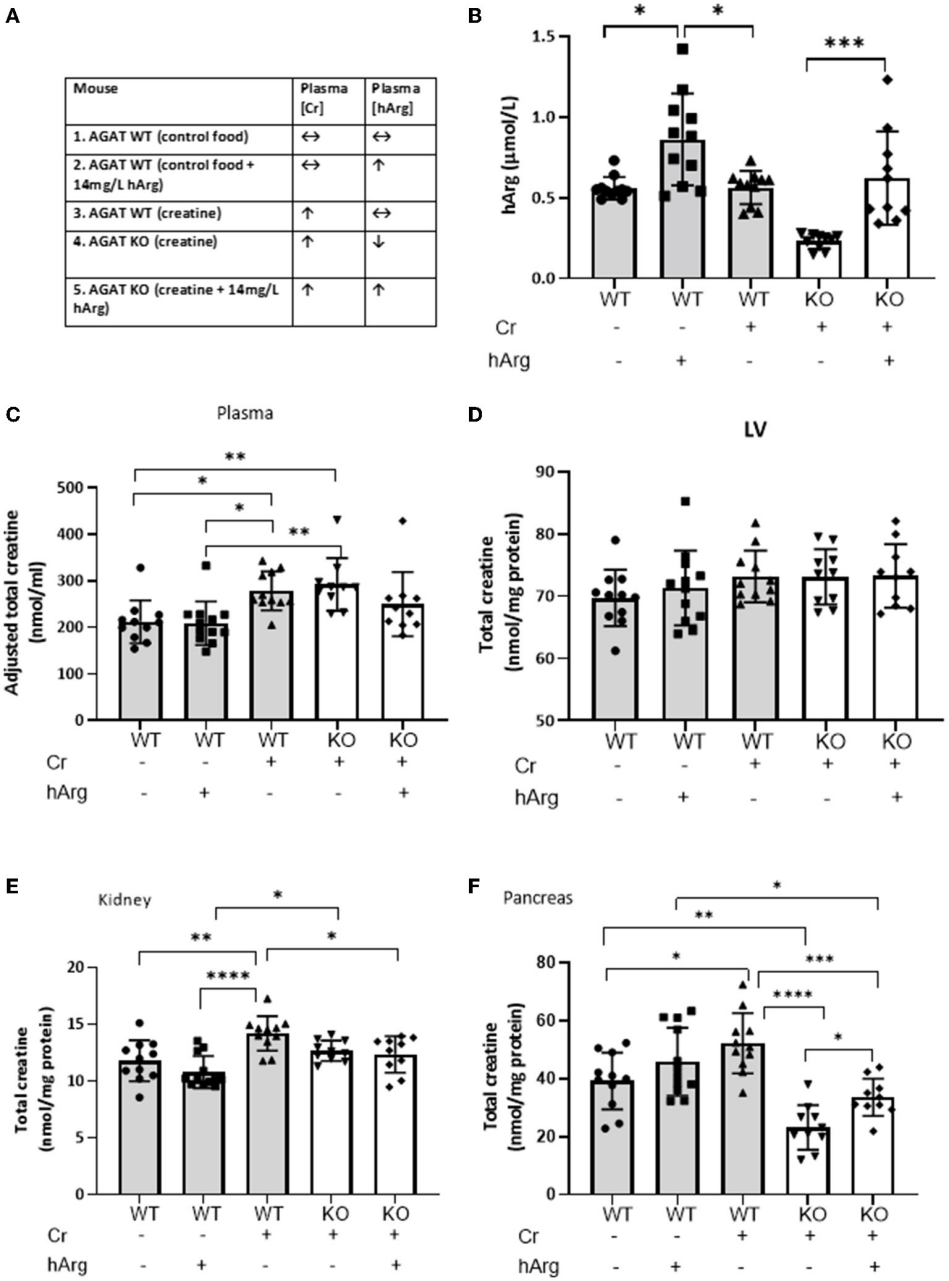
**(A)** Experimental groups describing dietary supplementation of either creatine of hArg or combined. **(B)** Levels of hArg in plasma of WT (gray columns) and KO (white columns) mice in the five experimental groups. Data analyzed by One-way ANOVA, *post-hoc* analysis Dunnet. *P<0.05; ***P<0.001. Data mean ± SEM shown per group. *n* = 11 for each WT group and n=10 per KO groups. Total creatine levels measured by HPLC in **(C)** plasma, **(D)** left ventricular tissue (LV), **(E)** kidney and **(F)** pancreas in mouse groups. (-) no feeding; (+) feeding. Gray columns: wild-type (WT); white columns: KO. Data showing cohorts of *n* = 10–12 and analyzed by one-way ANOVA, followed by multiple comparisons between different groups. *P < 0.05; **P < 0.01; ***P < 0.001; ****P < 0.0001.

We then sought to determine whether levels of hArg influence tissue creatine. Kidney and pancreas were selected since these were found to express AGAT, while the LV was included as a representative tissue with high creatine requirements, but which is wholly reliant on creatine-uptake. The various experimental groups with dietary supplementation regimes are shown in Figure 2A.

We demonstrate that hArg feeding increases levels of the metabolite in the plasma compared to untreated (Figure 2B, columns 1-3). KO (column 4) have lower basal hArg levels compared to WT confirming AGAT as the major source of hArg, whereas supplementation of hArg increases concentration in plasma (column 5). The presence or absence of creatine did not influence circulating levels of hArg. Plasma hArg in untreated KO was previously shown to be at 0.04 ± 0.02 vs hArg-supplemented KO at 0.60 ± 0.14 μmol/l, while WT littermates had 0.30 ± 0.16 μmol/l (18). In the same cohorts creatine values were reported as follows: WT: 92.0 ± 1.1 nmol/mg protein, unfed KO: 1.5 ± 0.6 nmol/mg protein.

As expected, creatine feeding increased plasma levels in both WT and KO compared to WT control animals by ~30% (Figure 2C, column 1 vs. columns 3 & 4; *P* < 0.05). This just failed to reach statistical significance when hArg was co-administered (column 5; *P* = 0.34), but could not reflect an effect on AGAT activity since these animals are AGAT deficient. Homoarginine supplementation did not affect circulating creatine levels (column 1 vs. 2), which argues against feedback inhibition of AGAT by hArg at the whole body level.

LV creatine levels were completely unaltered in response to either creatine feeding or hArg supplementation (Figure 2D). Expression data from Study 1 confirmed the absence of biosynthetic enzymes in LV and the reliance therefore of creatine-uptake *via* the CrT. Hence, it can be concluded that hArg supplementation does not influence cellular creatine uptake in the heart.

Dietary creatine increased creatine levels in the kidney by approximately 20% compared to WT controls (Figure 2E, column 1 vs. 3; *P* < 0.01), whereas hArg alone (column 2) did not alter creatine levels, again indicating that circulating hArg does not influence AGAT activity. Absence of AGAT protein in KO animals blunted the increase in kidney creatine, as shown by both the creatine alone and combination of creatine + hArg groups compared to untreated WT (columns 4 and 5 vs. column 1). This likely reflects the absence of endogenous creatine synthesis *via* AGAT, however the high levels of CrT expressed in the kidney (Figure 2E) are clearly sufficient to maintain normal creatine levels regardless.

Creatine supplementation increased intracellular levels in WT pancreas by over 30% compared to the untreated group (Figure 2F, column 3 vs. 1; *P* < 0.05). A trend toward increased creatine in the presence of hArg supplementation did not reach statistical significance (column 1 vs, 2; *P* = 0.34). AGAT deficiency lowered creatine levels by 40% compared to non-supplemented WT, despite dietary creatine supplementation (column 1 vs. 4; *P* < 0.01). Since pancreas is the only tissue that expressed both AGAT and GAMT (Figure 1), these findings suggest that pancreas relies on *de novo* creatine synthesis to a greater extent than creatine uptake *via* the CrT. In contrast to all other tissues, it is notable that creatine levels in KO animals were 30% higher when hArg was also supplemented (column 4 vs. 5; *P* = 0.018) without reaching WT levels. This result may suggest regulation of creatine uptake by hArg, which appears to be specific to the pancreas.

**Figure 3:**
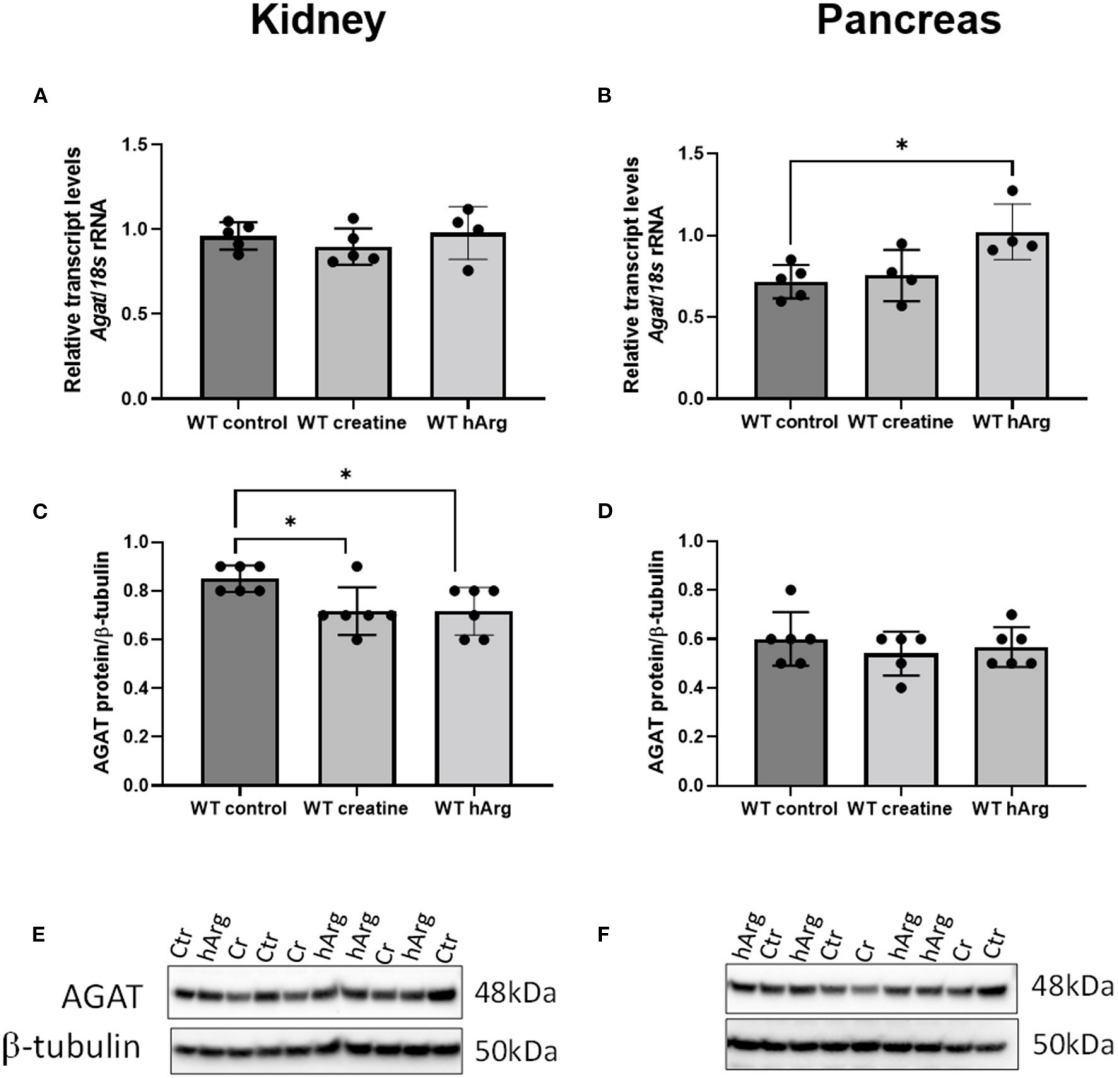
Effects of dietary creatine and hArg supplementation on AGAT transcript and protein levels in WT mice, in groups 1-3 (from Figure 2A). Kidney mRNA **(A)** and protein **(C)** levels. One-way ANOVA and Dunnett's *post-hoc* test of multiple comparisons vs. WT without creatine/hArg (control) *P = 0.03. Pancreatic AGAT mRNA **(B)** and protein **(D)**. *P = 0.018 for comparison between Ctr and hArg. Mean ± SEM is shown on graphs. Representative protein bands on immunoblots of AGAT protein from kidney **(E)** and pancreas **(F)** samples. Protein signal was detected at 48kDa for AGAT and at 50kDa for β-tubulin. Cr, creatine; hArg, homoarginine.

To detect any changes in *Agat* due to either dietary creatine or hArg reflecting end-product inhibition, qRT-PCR analyzed transcript in WT groups. Supplementation of creatine did not affect kidney or pancreatic *Agat* mRNA (Figure 3A). However, hArg caused a slight upregulation of *Agat* transcript in the pancreas (One-way ANOVA, *post-hoc* Dunnett test vs. WT untreated; *P* = 0.018) (Figure 3B) but not in kidney indicating that the biosynthetic enzyme may have a tissue specific function in pancreatic cells. When protein levels were assessed, kidney AGAT was subjected to end-product inhibition by both creatine and homoarginine (One-way ANOVA, *post-hoc* Dunnett test vs. WT untreated; *P* < 0.05) (Figures 3C,E) in contrast to pancreatic AGAT that is unaffected by dietary intervention using either creatine or homoarginine (Figures 3D,F).

## Discussion

In the current study we identify the potential for creatine biosynthesis in a range of mouse tissues by detection of transcript and protein levels of the biosynthetic enzymes AGAT (for creatine and hArg) and GAMT (for creatine). Our expression pattern is in agreement with published data for rats and humans (7, 35–37) confirming in the mouse, through a systematic study, that kidney and pancreas express AGAT, while liver and pancreas are the major sites of GAMT expression. This supports the concept of creatine biosynthesis as a two-step process in kidney and liver (4), but also that pancreas is capable of *de novo* creatine synthesis as previously reported in rat (5, 36). AGAT mRNA and protein are thought to be expressed in the mitochondria of acinar cells suggesting a role in exocrine pancreas function (38). However, the physiological requirement for creatine has not been established and it is unclear why the pancreas should have its own creatine supply when other tissues that are highly dependent on creatine (e.g., heart, skeletal muscle) do not. Other studies have shown that pancreatic AGAT levels are higher than GAMT (36) and we found CrT expression to be negligible, opening up the possibility that AGAT is expressed in pancreas to support local hArg synthesis. This suggests that the role of hArg on normal pancreatic function deserves further study.

CrT was strongly expressed in kidney, presumably for the purpose of creatine reabsorption from urine. Tissues that lacked the enzymes for *de novo* synthesis of creatine, also displayed substantial expression of creatine transporter, namely in skeletal and heart muscle, but also in BAT, indicating a reliance on creatine uptake rather than local synthesis.

Creatine has recently emerged as a regulator of energy expenditure and thermogenesis in both beige and brown adipose tissue as part of a “futile creatine cycle” catalyzed by B-type creatine kinase (20, 39). This process may represent a therapeutic target for the treatment of obesity since adipocyte-specific depletion of AGAT in mice exacerbated diet-induced obesity and glucose intolerance (21). Our data does not support the expression of AGAT in BAT and it is notable that in the study by Kazak et al. the residual adipose creatine remained relatively high (40–8% of WT). However, the same authors have since recapitulated their findings using mice with adipose-specific CrT deletion (40), which our data agrees is the more appropriate target for creatine manipulation in BAT.

It is a limitation that our current work does not include measurements from brain tissue, which has both the potential for local creatine biosynthesis and uptake of circulating creatine via expression of the CrT at the blood-brain barrier (41). However, several studies demonstrate the positive action of exogenous creatine on the brain for example, to improve cognitive processing (42, 43).

It should be noted that we did not measure guanidinoacetate and creatine production directly in tissues and can therefore only infer biosynthetic capacity. Furthermore, direct comparison between tissues is problematic since there can be large variations in housekeeping genes used for normalization. Our analysis should therefore be treated as qualitative rather than quantitative. Nevertheless, the agreement with published data provides reassurance that recent findings using mouse models will be broadly translatable to other mammalian species.

For the second part of this study, we adopted a unique experimental set-up making use of the AGAT KO mouse. Since standard chow is creatine-free, the only source of creatine is from supplementation or biosynthesis (available to WT but not KO animals). hArg is also absent from standard chow and by far the largest endogenous source is AGAT, with a small residual amount from lysine (instead of ornithine) entering the urea cycle (8). This was a successful strategy with dietary supplementation producing the desired effects on plasma levels of hArg and creatine.

As expected, dietary supplementation did not increase creatine levels in WT myocardium, but precisely replenished creatine stores in the KO hearts to WT levels. This reflects the absence of local biosynthetic capacity along with tight regulation of CrT activity in the heart. Creatine-deficiency is known to up-regulate the CrT (44), while elevated creatine results in down-regulation (45), likely *via* a negative feedback mechanism involving synthesis of an endogenous inhibitor, Txnip (32). Here we show for the first time that levels of hArg do not have any influence on myocardial creatine accumulation.

The effects of dietary manipulation in kidney and pancreas are more nuanced since both organs express AGAT, which can be subject to end-product repression (46). For example, creatine supplementation in rats reduced AGAT gene expression and activity in the kidneys to 37 and 26% of control levels, respectively (47). Similarly, after creatine feeding rats, da Silva et al. (7) describe a reduction in kidney AGAT mRNA of 47% and a drop in enzyme activity of 83%. These are large effect sizes that are not apparent in our data, where AGAT mRNA expression was completely unaffected by creatine supplementation. This is unlikely to be explained by differences in dietary creatine since we used 0.5% creatine compared to 0.3 and 0.4% by weight in earlier studies. This means species differences are a more likely explanation, with rats but not mice, exhibiting end-product repression of AGAT in the kidney, at least at the transcriptional level. Mice must surely have an alternative mechanism for down-regulation of creatine biosynthesis, since it is a costly process in terms of metabolic resources (5) and this merits further study.

It has previously been suggested that AGAT protein in the kidneys and the pancreas may be distinct based on small differences in tissue immunogenicity (48). This is also reflected in AGAT regulation, whereby in the same study of creatine supplementation discussed above, AGAT activity in the pancreas was only reduced by 34% (cf. 83% in kidney) with no change at all in mRNA and protein expression (7). Our results in mouse kidney also suggest a moderate but significant decrease of pancreatic AGAT with both creatine and homoarginine supplementation, which is consistent with the hypothesis of end-product inhibition by creatine and for the first time, by hArg. However, this was not observed in the pancreas, a unique finding that merits further study.

Another curious observation in the pancreas is that creatine supplementation in the AGAT KO animals increased tissue creatine levels, despite the absence of CrT expression. Furthermore, that creatine levels increased further in the presence of hArg, suggesting positive regulation of creatine uptake by hArg in this tissue. These results cannot be explained by changes in local creatine biosynthesis since the effect was most pronounced in KO animals. We cannot rule out that pancreas expresses alternative transporters capable of creatine uptake, but it could also simply reflect the high concentration gradient between plasma and tissue in supplemented KO animals that drives some limited diffusion across the plasma membrane.

This work shows tissue-specific expression of creatine biosynthetic enzymes AGAT and GAMT in addition to CrT and provides comparison to already published work in other species. Further metabolic analyses can provide understanding of whole-body utilization, especially if there is depletion of substrate or precursor amino acid availability. Future studies would benefit key organs such as the pancreas, where biosynthesis of creatine could be relevant to local physiology in addition to modulating energy homeostasis in other organs.

## Data availability statement

The original contributions presented in the study are included in the article/supplementary material, further inquiries can be directed to the corresponding authors.

## Ethics statement

The animal study was reviewed and approved by the Committee for Animal Care and Ethical Review at the University of Oxford and comply with the Animals (Scientific Procedures) Act 1986.

## Author contributions

Study design, supervision, and manuscript preparation: CL and SZ. Experimental work: HL, DM, and SZ. Manuscript proofing and editing: CL, SZ, HL, DM, and SN. Funding: CL and SN. All authors contributed to the article and approved the submitted version.

## Funding

This work was supported by British Heart Foundation Programme Grants (RG/13/8/30266 and RG/18/12/34040) to SN and CL. Additional core support was acknowledged from the Oxford BHF Center for Research Excellence and Wellcome Trust Core Award (Grant No. 203141/Z/16/Z).

## Conflict of interest

The authors declare that the research was conducted in the absence of any commercial or financial relationships that could be construed as a potential conflict of interest.

## Publisher's note

All claims expressed in this article are solely those of the authors and do not necessarily represent those of their affiliated organizations, or those of the publisher, the editors and the reviewers. Any product that may be evaluated in this article, or claim that may be made by its manufacturer, is not guaranteed or endorsed by the publisher.
